# Supplementary material for: Augmin prevents merotelic attachments by promoting proper arrangement of bridging and kinetochore fibers
Source: eLife. 2022 Oct 21;11:e83287. doi: 10.7554/eLife.83287 (PMC9640188; doi:10.7554/eLife.83287)
Supplement: Figure 2—source data 1. — Full unedited blots of all three independent experiments are shown. [file elife-83287-fig2-data1.pdf]

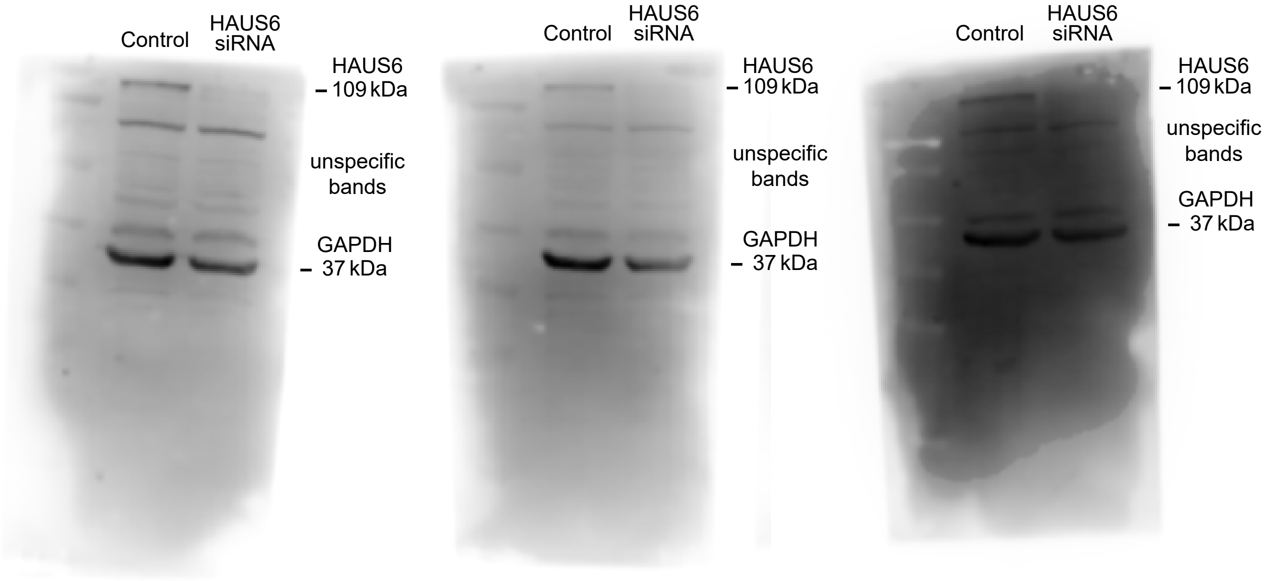

Control
HAUS6 siRNA
HAUS6
109 kDa
unspecific bands
GAPDH
37 kDa
Control
HAUS6 siRNA
HAUS6
109 kDa
unspecific bands
GAPDH
37 kDa
Control
HAUS6 siRNA
HAUS6
109 kDa
unspecific bands
GAPDH
37 kDa
